# Factors associated with neonatal near miss in Brazil

**DOI:** 10.11606/s1518-8787.2020054002382

**Published:** 2020-11-23

**Authors:** Theonas Gomes Pereira, Daniele Marano da Rocha, Vânia Matos Fonseca, Maria Elisabeth Lopes Moreira, Silvana Granado Nogueira da Gama

**Affiliations:** I Centro Universitário UNINOVAFAPI TeresinaPI Brasil Centro Universitário UNINOVAFAPI. Curso de Nutrição. Teresina, PI, Brasil; II Fundação Oswaldo Cruz Instituto Nacional de Saúde da Mulher, da Criança e do Adolescente Fernandes Figueira Rio de JaneiroRJ Brasil Fundação Oswaldo Cruz. Instituto Nacional de Saúde da Mulher, da Criança e do Adolescente Fernandes Figueira. Pós-Graduação em Saúde da Criança e da Mulher. Rio de Janeiro, RJ, Brasil; III Fundação Oswaldo Cruz Escola Nacional de Saúde Pública Departamento de Epidemiologia em Métodos Quantitativos em Saúde Rio de janeiroRJ Brasil Fundação Oswaldo Cruz. Escola Nacional de Saúde Pública. Departamento de Epidemiologia em Métodos Quantitativos em Saúde. Rio de janeiro, RJ, Brasil

**Keywords:** Near Miss, Healthcare, Pregnancy Complications, Risk Factors, Socioeconomic Factors, Maternal-Child Health Services

## Abstract

**OBJECTIVE:**

This study evaluates the association between sociodemographic factors, maternal characteristics, organization of health services and neonatal near miss in public and private maternity hospitals in Brazil.

**METHODS:**

This is a prospective cohort of live births from the *Nascer no Brasil* survey, carried out between 2011 and 2012. Variables were established from the literature and organized on three levels: distal, intermediate, and proximal. The assessment was performed based on results of the bivariate analyzes and their respective p-values, with a significance level <0.20, using the Wald test. For multivariate analysis, the variables contained at the distal level were inserted, preserved in the model when significant (p < 0.05). This was also done when adjusting the intermediate and proximal levels.

**RESULTS:**

At the distal level, no variable was significantly associated with the outcome. At the intermediate level, mother's age greater than or equal to 35 years (relative risk – RR = 1.32; 95%CI 1.04–1.66), cesarean delivery (RR = 1.34; 95%CI 1.07–1.67), smoking (RR = 1.48; 95%CI 1.04–2.10), gestational hypertensive syndrome (RR = 2.29; 95%CI 1.98–3.14), pre-gestational diabetes (RR = 2.63; 95%CI 1.36–5.05) and twin pregnancy (RR = 2.98; 95%CI 1.90–4.68) were variables associated with the outcome. At the proximal level, inadequate prenatal care (RR = 1.71; 95%CI 1.36–2.16) and the hospital/maternity being located in a capital city (RR = 1.89; 95%CI 1.40–2.55) were associated with neonatal near miss.

**CONCLUSIONS:**

The results show that neonatal near miss was influenced by variables related to the organization of health services and by maternal characteristics.

## INTRODUCTION

The concept of neonatal near miss is recent, being defined as morbid events that almost result in death of newborns (NB) in the first 28 days of life^1^^,^^2^. Since there are different definitions in the literature for neonatal near miss^2^^–^^4^, this study used the concept adopted by Silva *et al*.^2^, who, in 2014, evaluated data from the *Nascer no Brasil* survey – a national hospital-based study at the regional level – in order to define variables that could predict neonatal mortality and compose the neonatal near miss indicator. After 19 variables were tested, 5 were chosen, namely: birth weight < 1,500g, Apgar < 7 in the fifth minute of life, use of mechanical ventilation (MV), gestational age of < 32 weeks, and report of congenital malformations. The authors of the aforementioned study^2^ assessed that this indicator has high sensitivity (92.5%), specificity (97.1%) and accuracy (97%), which gives strength to its use and the monitoring of this condition.

The criteria defined by Silva *et al*.^2^ were validated by the studies conducted by Kale *et al*. ^3^ and França *et al*.^4^ In the first study^3^, a cohort of live births in two Brazilian capitals, three pragmatic criteria were used by Silva *et al*. ^2^ to define neonatal near miss: birth weight < 1,500g, gestational age of < 32 weeks, and Apgar score < 7 in the fifth minute of life. In the second study^4^, also a cohort of live births, data from the Health Information Systems were used, selecting the variables used in the study by Kale *et al*.^3^, plus admission to the neonatal intensive care unit (ICU) and congenital malformations. Both studies showed the accuracy of the proposal by Silva *et al*.^2^

The neonatal near miss indicator offers numerous advantages, as it is a tool to identify risk factors associated with neonatal death and to monitor changes in neonatal morbidity and mortality^5^. Among these advantages, one can mention the identification of serious morbidities and their primary causes, which can reduce neonatal death and allows for the indicator to be used in several configurations to identify problems in the health system – becoming a management tool – and, if applicable, to take corrective actions^2^, leading to an improved quality of neonatal care^5^.

Regarding infant deaths in Brazil, it is observed that this outcome occurs mainly in the neonatal period (70%), especially in the first weeks of life (54%)^6^. Therefore, there is a reduction in infant mortality in the post-neonatal period (from 23.1 to 9.5 per thousand live births)^6^.

Hence, several authors have discussed neonatal mortality^6^^,^^7^; however, there are few studies that have analyzed the main factors associated with neonatal near miss^5^^,^^8^. Advancement in the knowledge of the network of maternal risk factors involved in neonatal mortality (age, education^6^, marital status, smoking and use of alcohol^9^, previous and current diseases of pregnancy, adequacy of prenatal care, among others)^10^, based on the hierarchical modeling strategy to discriminate the relationships between neonatal near miss determinants, can be useful in its evaluation; moreover, it enables us to indicate actions necessary to improve care, with a consequent impact on neonatal outcomes.

Therefore, this study aims to assess the association between sociodemographic factors, maternal characteristics, the organization of health services and neonatal near miss in public and private hospitals, representative of the five regions of Brazil.

## METHODS

This research is a prospective cohort of live births, consisting of information from the questionnaires applied to the puerperal women and data collected from the medical records of patients who participated in the *Nascer no Brasil* survey. Data collection took place between February 2011 and October 2012. Details regarding sampling are found in the study by Vasconcellos *et al*.^11^ and, on the method, in Leal *et al*.^12^

For the construction of the dependent variable of this study, the neonatal near miss, the classification of the study by Silva *et al*.^2^ was used, which selected five variables associated with neonatal mortality: birth weight < 1,500g, Apgar score < 7 in the fifth minute of life, use of MV, gestational age < 32 weeks, and presence of congenital malformations. Thus, all newborns who survived the neonatal period and had at least one of the mentioned predictors were considered cases of neonatal near miss^2^.

24,200 newborns were sampled, 23,837 of whom were born alive, 128 stillborn, 171 neonatal deaths and 64 neonatal deaths rescued from the *Sistema de Informações sobre Mortalidade* (SIM – Mortality Information System). The cases of neonatal deaths after hospital discharge were obtained through a questionnaire applied after the 42nd day of hospitalization of the woman or on the 28th day of hospitalization of the newborn. More detailed information about the method can be obtained in the study by Silva *et al*^2^.

The hierarchical model of neonatal near miss was based on risk factors for the NB death^13^. It is noteworthy that the health conditions of newborns and neonatal care are inherent to the definition of neonatal near miss (gestational age, birth weight, Apgar score, among others). Therefore, variables related to the organization of the health service were considered at the proximal level. Independent variables were organized by level of proximity to the outcome, first inserting those at the distal level and then those at the intermediate and proximal levels, established from the literature^1^^,^^2^^,^^6^^,^^8^ and organized in a theoretical-conceptual model (Figure).

**Figure:**
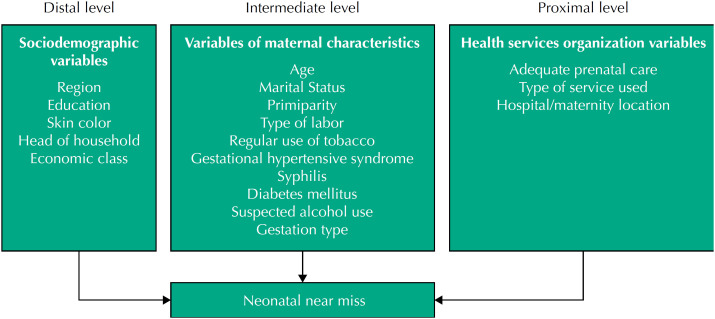
Theoretical-conceptual model of predictive factors for neonatal near miss in Brazil.

At the distal level, sociodemographic aspects were included: region (Southeast, North, Northeast, Central-West or South); maternal education in complete years (incomplete elementary school, complete elementary school, complete high school or complete higher education); economic class (A + B, C, or D + E); skin color (white or black/brown/yellow/indigenous); and head of household (no or yes). Mothers were considered “head of household” when she was the reference person for decision-making in the family^11^, and the economic classification was based on the criteria of the Brazilian Association of Research Companies (Abep)^14^.

At the intermediate level, variables representative of maternal characteristics were included: maternal age (12 to 19 years, 20 to 34 years, or greater than or equal to 35 years); marital status (without a partner or with a partner); primiparity (no or yes); type of labor (vaginal, with forceps or cesarean); maternal smoking, considering the regular use of tobacco after the fifth month of pregnancy (no or yes); hypertensive pregnancy syndrome (no or yes); syphilis (no or yes); pre-gestational diabetes (no or yes); gestational diabetes (no or yes); suspicion of inappropriate alcohol use (no or yes); and type of pregnancy (single or twin). To measure smoking, the variable smoking after the fifth month of pregnancy was considered to be at greater risk for low weight in NB^15^. As for the suspicion of alcohol use, the T-ACE questionnaire (acronym of the English words: *tolerance*, annoyed, *cut down* and *eye-opener*) was used, composed of four main questions, to which a score is attributed, being the maximum value equal to five (the first question is worth up to two points and, from the second to the fourth question, the rating is up to one point). A total score greater than or equal to two indicates a positive case, that is, the mother is identified as an alcohol consumer^16^. The gestational hypertensive syndrome variable refers to the diagnosis of chronic hypertension, gestational hypertension, pre-eclampsia, eclampsia or Hellp syndrome^17^.

At the proximal level, variables related to the organization of the health service were considered: adequate prenatal care (no or yes); type of service used in prenatal care (public or private); and location of the hospital/maternity (not in a capital city or capital city). Adequate prenatal care was considered to be that started until the 12th gestational week, with at least six consultations (value corrected according to gestational age at the time of delivery), recording on the prenatal card of at least one result of each exam routine and receiving guidance for a reference maternity^10^.

The variable gender of the newborn (male or female) was not included in any level of hierarchical determination; however, it was part of the final model because it is an important predictor of neonatal mortality^7^.

For data analysis, initially, the absolute and relative frequencies of the predictor variables were estimated. The bivariate analysis used Pearson's chi-square test, relative risk (RR) and 95% confidence intervals (CI) to assess the association of variables. Multivariate analysis used Poisson regression models with robust variance to identify the variables associated with neonatal near miss. The RR was used to analyze the association of sociodemographic, maternal and health service organization variables with neonatal near miss. Variables with a p-value < 0.20 in the bivariate analysis were selected for multivariate analysis. Only variables with a p-value < 0.05 in the multivariate model were maintained in the final model. Collinear variables with a variance inflation factor < 10 were excluded from the model.

The main study was approved by the Research Ethics Committee (REC) of the National School of Public Health of the Oswaldo Cruz Foundation (Opinion No. 92/10; CAE: 0096.0.031.000-10). This research was submitted to the REC of the National Institute of Health of Women, Children and Adolescents Fernandes Figueira and approved under Opinion No. 3.376.235 (CAAE: 14248719.1.0000.5269), fulfilling the precepts of Resolution No. 466/2012 of the National Health Council^18^. All participants gave their interviews and information through a free and informed consent form.

## RESULTS

In this research, 832 was the weighted number of NB who met the neonatal near miss criteria, and 23,005 did not, totaling 23,837 newborns. Table 1 shows that the risk of occurrence of neonatal near miss, when comparing the sociodemographic categories, was higher among women who had incomplete primary education (4.2%), who declared themselves black/brown/yellow/indigenous (4.1%) and belonging to class C (3.8%).

**Table 1:** Distribution of sociodemographic conditions (distal level) regarding neonatal near miss. Brazil, 2011–2012.

Variables		% Total	% NNM	RR	95%CI	p^a^
Sex						
	Male	51.7	4.1	1.15	0.98–1.35	0.078
	Female	48.3	3.5	1	–	–
Distal						
	Region					
	Southeast	42.6	4.3	1.38	0.99–1.92	0.057
	North	9.5	3.1	0.99	0.63–1.56	0.980
	Northeast	28.8	3.5	1.12	0.74–1.69	0.592
	South	12.5	3.1	1	–	–
	Central-West	6.6	4.1	1.31	0.86–2.01	0.204
Education						
	Incomplete primary school	26.5	4.2	1.18	0.82–1.69	0.357
	Complete primary school	25.6	3.6	1.03	0.69–1.52	0.875
	Complete high school	38.9	3.7	1.04	0.68–1.59	0.837
	Complete higher education or above	8.9	3.5	1	–	–
Ethnicity/color						
	White	33.8	3.3	1	–	–
	Black/brown/yellow/indigenous	66.2	4.1	1.24	0.98–1.56	0.075
Head of household				
	No	89.6	3.9	1	–	–
	Yes	10.4	3.0	0.76	0.57–1.02	0.071
Economic class^b^						
	Class D+E	23.6	3.8	1.14	0.89–1.47	0.303
	Class C	52.0	4.0	1.18	0.89–1.56	0.249
	Class A+B	24.3	3.4	1	–	–

NNM: neonatal near miss; RR: relative risk; 95%CI: 95% confidence interval.

a P-value: Pearson's chi-square test.

b According to Abep classification.

As for maternal characteristics (Table 2), the risk of near miss was higher among women who had a cesarean delivery (4.3%), who reported using tobacco (5.1%), who had hypertensive pregnancy syndrome (8.7%), pre-gestational (12%) and gestational (4.4%) diabetes. In addition, a greater risk of neonatal near miss was observed in women with twin pregnancies (11.8%), when compared to those with single pregnancies (3.7%).

**Table 2:** Distribution of maternal characteristics (intermediate level) regarding neonatal near miss. Brazil, 2011–2012.

Variables		% Total	% NNM	RR	95%CI	p*
Sex						
	Male	51.7	4.1	1.15	0.98–1.35	0.078
	Female	48.3	3.5	1	–	–
Intermediate						
	Age (years)					
	12 to 19	19.1	4.3	1.24	0.95–1.62	0.107
	20 to 34	70.5	3.4	1	–	–
	≥ 35	10.4	5.2	1.51	1.23–1.85	< 0.001
Marital Status						
	Without partner	18.5	4.3	1.18	0.94–1.46	0.138
	With partner	81.5	3.7	1	–	–
Primiparous						
	No	53.3	3.6	1	–	–
	Yes	46.7	4.0	1.12	0.94–1.35	0.207
Type of labor						
	Vaginal birth	46.6	3.2	1	–	–
	Forceps	1.4	5.4	1.70	0.88–3.31	0.115
	Cesarean section	52.0	4.3	1.36	1.10–1.67	0.004
Regular use of tobacco					
	No	92.8	3.7	1	–	–
	Yes	7.2	5.1	1.38	0.97–1.96	0.069
Gestational hypertensive syndromes						
	No	89.0	3.2	1	–	–
	Yes	11.0	8.7	2.71	2.21–3.33	< 0.001
Syphilis						
	No	99.0	3.8	1	–	–
	Yes	1.0	4.9	1.31	0.65–2.64	0.448
Pre-gestational diabetes						
	No	99.0	3.7	1	–	–
	Yes	1.0	12.0	3.23	1.9–5.3	< 0.001
Gestational diabetes						
	No	91.8	3.8	1	–	–
	Yes	8.2	4.4	1.18	0.92–1.52	0.197
Suspected misuse of alcohol						
	There is no suspicion	3.9	4.3	1.16	0.66–2.03	0.609
	Suspected use	10.0	4.5	1.23	0.94–1.61	0.126
Did not drink alcohol		86.1	3.7	1	–	–
Gestation type						
	Single	98.8	3.7	1	–	–
	Twin	1.2	11.8	3.18	2.25–4.50	< 0.001

NNM: neonatal near miss; RR: relative risk; 95%CI: 95% confidence interval.

* P-value: Pearson's chi-square test.

In the block referring to the organization of health services (Table 3), a greater risk of neonatal near miss was identified in the children of women who did not receive adequate prenatal care. In addition, this outcome was higher (5.5%) when delivery occurred in a capital city.

**Table 3:** Distribution of the health service organization (proximal level) regarding neonatal near miss. Brazil, 2011–2012.

Variables		% Total	% NNM	RR	95%CI	p*
Sex						
	Male	51.7	4.1	1.15	0.98–1.35	0.078
	Female	48.3	3.5	1	–	–
Proximal						
Adequate prenatal care						
	No	36.7	5.1	1.66	1.32–2.08	< 0.001
	Yes	63.3	3.1	1	–	–
Place of prenatal consultations						
	Public	70.7	4.0	1.27	1.00–1.62	0.050
	Private	29.3	3.1	1	–	–
Hospital/maternity location						
	Not in a capital city	63.4	2.8	1	–	–
	Capital city	36.6	5.5	1.95	1.44–2.65	< 0.001

NNM: neonatal near miss; RR: relative risk; 95%CI: 95% confidence interval.

* P-value: Pearson's chi-square test.

Tables 1, 2 and 3 show the results of the bivariate analysis for all independent variables included in the model. At the distal level (Table 1), no variable was associated with the outcome. At the intermediate level (Table 2), the following variables were associated with neonatal near miss: age greater than or equal to 35 years (RR = 1.51; 95%CI 1.23–1.85), cesarean delivery (RR = 1.36; 95%CI 1.10–1.67), gestational hypertensive syndrome (RR = 2.71; 95% CI 2.21–3.33), pre-gestational diabetes (RR = 3.23; 95%CI 1.90–5.30) and twin pregnancy (RR = 3.18; 95%CI 2.25–4.50). There was no association between gestational diabetes *mellitus* and neonatal near miss. At the proximal level (Table 3), the following variables were associated: inadequate prenatal care (RR = 1.66; 95%CI 1.32–2.08) and delivery in the capital (RR = 1.95; 95%CI % 1.44–2.65).

Table 4 shows the multivariate regression model. There was a significant association between neonatal near miss and the following variables: mother's age greater than or equal to 35 years (RR = 1.32; 95%CI 1.04–1.66), cesarean delivery (RR = 1.34; 95%CI 1.07–1.67), habitual use of tobacco (RR = 1.48; 95%CI 1.04–2.10), hypertensive pregnancy syndrome (RR = 2.49; 95%CI 1.98 –3.14), pre-gestational diabetes (RR = 2.63; 95%CI 1.36-5.05), twin pregnancy (RR = 2.98; 95%CI 1.90–4.68), inadequate prenatal care (RR = 1.71; 95%CI 1.36–2.16) and location of the hospital/maternity in the capital (RR = 1.89; 95%CI 1.40–2.55).

**Table 4:** Multivariate regression of sociodemographic conditions. maternal characteristics and the organization of health services regarding neonatal near miss. Brazil, 2011–2012.

Variables		Adjusted RR	95%CI	p*
Sex of newborn				
	Male	1.18	1.00–1.40	0.054
	Female	1	–	–
Distal				
Ethnicity/color				
	White	1	–	–
	Black/brown/yellow/indigenous	1.21	0.95–1.55	0.126
Intermediate				
Age (years)				
	12 to 19	1.28	0.98–1.67	0.073
	20 to 34	1	–	–
	≥ 35	1.32	1.04–1.66	0.020
Type of labor				
	Vaginal birth	1	–	–
	Forceps	1.75	0.81–3.77	0.151
	Cesarean section	1.34	1.07–1.67	0.009
Regular use of tobacco				
	No	1	–	–
	Yes	1.48	1.04–2.10	0.031
Gestational hypertensive syndromes				
	No	1	–	–
	Yes	2.49	1.98–3.14	< 0.001
Pre-gestational diabetes				
	No	1		
	Yes	2.63	1.36–5.05	0.004
Gestation type				
	Single	1	–	–
	Twin	2.98	1.90–4.68	< 0.001
Proximal				
Adequate prenatal care				
	No	1.71	1.36–2.16	< 0.001
	Yes	1	–	–
Hospital/maternity location				
	Not in a capital city	1	–	–
	Capital city	1.89	1.40–2.55	< 0.001

RR: relative risk; 95%CI: 95% confidence interval.

* P-value: Pearson's chi-square test.

## DISCUSSION

The results revealed the prominence of maternal characteristics (intermediate level) in the determination of neonatal near miss, with an important contribution from the care conditions received in prenatal care (proximal level), all considered factors that are likely to intervene^10^.

The maternal age group equal to or greater than 35 years, considered a risk factor for numerous negative outcomes related to NB^19^^,^^20^, had its association with neonatal near miss confirmed in this research, corroborating other studies in the literature^2^^,^^8^. The prospective birth cohort study in six Brazilian maternity hospitals conducted by Kale *et al*.^8^ observed that newborns of mothers with advanced maternal age had almost twice the risk of neonatal near miss. Women older than 35 years old have a higher frequency of adverse perinatal results when compared to women aged 20 to 34 years, with emphasis on prematurity, low birth weight, and low Apgar score^20^. In addition, the children of these women are at greater risk of dying in the neonatal period due to obstetric complications secondary to pre-existing diseases^21^.

Cesarean delivery remained associated with the occurrence of neonatal near miss in this study, a result already indicated in the literature in the area^2^^,^^5^^,^^8^. Silva *et al*.^2^, also in the *Nascer no Brasil* survey, observed that the chance of neonatal near miss was twice as high among women who underwent cesarean sections; i.e., this variable appears as a risk factor for such an outcome, but also as a protective factor for neonatal mortality, given that children born by vaginal delivery had a higher neonatal mortality rate. Thus, the mode of delivery itself would not cause maternal-fetal complications, but the clinical indication for cesarean section. To elucidate this point, it would be necessary to investigate whether the indication for cesarean section was intrapartum, due to maternal-fetal complications, or elective, without any clinical basis^5^.

The application of the hierarchical model in this investigation showed that, among the maternal factors analyzed, the habitual use of tobacco after the fifth month of pregnancy was associated with an increased risk of neonatal near miss. The adverse effects of maternal smoking during pregnancy affect the weight of the newborn. However, smoking is one of the most important modifiable determinants to minimize the risk of low birth weight and other adverse perinatal outcomes^15^. The negative impact of maternal smoking during the entire pregnancy on the newborn's length and head circumference indicates that such behavior has an inverse linear relationship with these dimensions: the longer the gestation period with exposure to smoke, the lower the anthropometric measurements of the NB^22^. These findings were pointed out in a population-based cohort of 8,621 European live births, in which it was observed that, from the beginning of the second trimester to the end of pregnancy, the fetuses of women who continued to smoke weighed less than those of non-smokers. More specifically, the expected weight difference in the children of women who smoked in the 20th week (95%CI) was −2.6g (-5.1 to −0.1), and in the 40th gestational week it was −207g (-231 to −182)^15^.

Regarding chronic diseases, it was observed that women with gestational hypertensive syndrome had twice the risk of neonatal near miss. Similarly, Oliveira *et al*.^23^ and Nardello *et al*.^19^, in cross-sectional studies in maternity hospitals in Recife and Sergipe, respectively, observed that gestational hypertension was strongly associated with adverse neonatal outcomes. Despite numerous factors and theories suggested to explain the possible causes of this condition, the etiology of gestational hypertensive syndrome is still poorly known^17^^,^^24^; however, its effects have been associated with prematurity, low Apgar and neonatal asphyxia^25^.

In the same line of reasoning, an association between pre-gestational diabetes mellitus and neonatal near miss was also observed. The increase in the prevalence of pre-gestational and gestational diabetes mellitus in recent years can be justified by the obesity epidemic, the increase in maternal age, and the early detection of the disease, considering the greater coverage of prenatal care and the decrease in the cutoff point diagnosis of gestational diabetes (fasting blood glucose reduced from 92mg/dL to 85mg/dL)^26^. As well as the gestational hypertensive syndrome, the presence of diabetes *mellitus* during pregnancy is also associated with a high risk of neonatal morbidity and mortality^27^. Some studies^25^^,^^27^ focused on the assessment of the association between pre-gestational diabetes and some neonatal outcomes, especially prematurity, congenital anomalies – such as cardiovascular malformations –, perinatal asphyxia, respiratory distress and metabolic complications (hypoglycemia, hypocalcemia, polycythemia and hyperbilirubinemia). Although these studies have not evaluated the effect of the disease on neonatal near miss, the aforementioned negative repercussions of pre-gestational diabetes mellitus on the health of NB show a possible elucidation of its effects on this outcome.

There was an approximately three times greater risk of neonatal near miss for twin pregnancies in this study. This result reveals that twin birth – a rare condition that presents several peculiarities and difficulties, not only in clinical management, but also in the scientific approach – is still considered a challenge for the health service and for investigations on greater maternal and perinatal risks^28^. It is worth mentioning that twin pregnancy increases the perinatal mortality rate by two to three times, mainly due to premature birth, intrauterine growth restriction, low birth weight and intrapartum anoxia^29^^,^^30^. Therefore, it is extremely important that there is adequate prenatal care aiming at better maternal and perinatal outcomes in this condition.

The lack of access and the quality of prenatal care are notable determinants for the occurrence of neonatal near miss^5^^,^^8^. In this study, the lack of adequacy of prenatal care (proximal level) was associated with this outcome, increasing its risk. Although prenatal care in Brazil has achieved practically universal coverage, inequalities in access to adequate care persist^10^^,^^11^. It is noteworthy that prenatal care enables the early detection and treatment of pre-existing maternal conditions and/or started during the gestational period, as well as changes in the conceptus, reducing the risk of obstetric complications and neonatal death due to prematurity, malformations or congenital infections, which are the most frequent causes of neonatal death in the world^9^^,^^10^. It is worth noting that the adequate number of prenatal consultations (six or more) does not guarantee in itself the quality of maternal and child care, and it is necessary to ensure the early start of prenatal care (up to the 12th week of pregnancy), assistance by qualified professionals, the existence of adequate physical and material resources, the performance of the recommended exams and the timely treatment, if necessary^17^^,^^21^^,^^25^^,^^26^.

Regarding the location of the hospital/maternity for delivery, it was observed that the risk of neonatal near miss almost doubled among NB who were born in the capitals. This result can be partially explained by the fact that non-capitals have a lower offer of specialized services for high-risk care^2^, with less suitable conditions for the care of pregnant women in this context. Thus, the capitals are a reference for pregnant women living in non-capitals that have complications in pregnancy^10^. It is noteworthy that the severity of the disease seems to be a confounding factor in the association between hospital of birth and neonatal near miss. The high availability of neonatal ICUs and the early medical intervention in large urban centers are factors pointed out by Silva *et al*.^2^ as possible justifications for the greater occurrence of neonatal near miss in the capitals. The present study did not aim to analyze the severity of neonatal near miss cases, thus making it impossible to point out whether the situation of NB worsened before or after treatment.

The main limitation of the study was the fact that it did not consider hospitals with less than 500 births and those born at home. It is noteworthy that the start of a second version of the *Nascer no Brasil* survey is scheduled for 2020, but so far no articles with neonatal near miss data have been found at the national level.

However, this study has the advantage of having been carried out from a hospital database representative of the Brazilian population. In addition, it offers a hierarchical analysis of the determination of neonatal near miss, with a wide range of variables for assessing the health of Brazilian pregnant women, allowing to analyze the interrelations involved in the causality network of this outcome. Therefore, the identification of the variables that have the greatest impact on the occurrence of neonatal near miss enables the adoption of preventive and intervention measures in the prenatal care of pregnant women, affecting the health of their newborns.

## CONCLUSIONS

Although characteristics subject to intervention by counseling – such as the regular use of tobacco – have been associated with the occurrence of neonatal near miss, other factors determining this outcome referred to the provision of services and care. Therefore, it is of utmost importance to emphasize the adequacy of prenatal care for the identification of pregnant women who need more specialized care, with timely monitoring during pregnancy, childbirth and the postpartum period to prevent life threatening perinatal conditions.
