# Supplementary material for: Factors associated with neonatal near miss in Brazil
Source: Rev Saude Publica. 2020 Nov 23;54:123. doi: 10.11606/s1518-8787.2020054002382 (PMC7688256; doi:10.11606/s1518-8787.2020054002382)
Supplement: Translation

# Translation: Fatores associados ao near miss neonatal no Brasil

**Authors:** Theonas Gomes Pereira, Daniele Marano da Rocha, Vânia Matos Fonseca, Maria Elisabeth Lopes Moreira, Silvana Granado Nogueira da Gama

**Affiliations:** I TeresinaPI Brasil Centro Universitário UNINOVAFAPI. Curso de Nutrição. Teresina, PI, Brasil; II Rio de JaneiroRJ Brasil Fundação Oswaldo Cruz. Instituto Nacional de Saúde da Mulher, da Criança e do Adolescente Fernandes Figueira. Pós-Graduação em Saúde da Criança e da Mulher. Rio de Janeiro, RJ, Brasil; III Rio de janeiroRJ Brasil Fundação Oswaldo Cruz. Escola Nacional de Saúde Pública. Departamento de Epidemiologia em Métodos Quantitativos em Saúde. Rio de janeiro, RJ, Brasil

**Keywords:** Near miss, Complicações na Gravidez, Fatores de Risco, Fatores Socioeconômicos, Serviços de Saúde Materno-Infantil

## Abstract

**OBJETIVO:**

Este estudo avalia a associação entre fatores sociodemográficos, características maternas, organização dos serviços de saúde e *near miss* neonatal em maternidades públicas e privadas do Brasil.

**MÉTODOS:**

Trata-se de uma coorte prospectiva de nascidos vivos da pesquisa *Nascer no Brasil*, realizada entre 2011 e 2012. As variáveis foram estabelecidas a partir da literatura e organizadas em três níveis: distal, intermediário e proximal. A avaliação foi realizada a partir dos resultados das análises bivariadas e de seus respectivos valores-p, com nível de significância < 0,20, pelo teste de Wald. Para a análise multivariada, foram inseridas as variáveis contidas no nível distal, conservadas no modelo quando significativas (p < 0,05). O mesmo foi feito no ajuste dos níveis intermediário e proximal.

**RESULTADOS:**

No nível distal, nenhuma variável apresentou associação significativa com o desfecho. No nível intermediário, idade da mãe maior ou igual a 35 anos (risco relativo – RR = 1,32; IC95% 1,04–1,66), parto cesáreo (RR = 1,34; IC95% 1,07–1,67), uso de fumo (RR = 1,48; IC95% 1,04–2,10), síndrome hipertensiva gestacional (RR = 2,29; IC95% 1,98–3,14), diabetes pré-gestacional (RR = 2,63; IC95% 1,36–5,05) e gestação gemelar (RR = 2,98; IC95% 1,90–4,68) foram variáveis associadas ao desfecho. No nível proximal, o pré-natal não adequado (RR = 1,71; IC95% 1,36–2,16) e a localização do hospital/maternidade na capital (RR = 1,89; IC95% 1,40–2,55) foram associados ao *near miss* neonatal.

**CONCLUSÃO:**

Os resultados mostram que o *near miss* neonatal foi influenciado pelas variáveis da organização dos serviços de saúde e pelas características maternas.

## INTRODUÇÃO

O conceito de *near miss* neonatal é recente, sendo definido como eventos mórbidos que quase resultam em morte de recém-nascidos (RN) nos primeiros 28 dias de vida^1^^,^^2^. Posto que na literatura existem distintas definições para o *near miss* neonatal^2^^–^^4^, este estudo considerou o conceito adotado por Silva *et al*.^2^, que, no ano de 2014, avaliaram os dados da pesquisa *Nascer no Brasil* – um estudo nacional de base hospitalar e nível regional – com o objetivo de definir as variáveis que poderiam predizer a mortalidade neonatal e compor o indicador *near miss* neonatal. Após serem testadas 19 variáveis, 5 foram escolhidas, a saber: peso ao nascer < 1.500g, Apgar no quinto minuto de vida < 7, uso de ventilação mecânica (VM), idade gestacional < 32 semanas e relato de más-formações congênitas. Os autores do estudo supracitado^2^ avaliaram que esse indicador tem elevada sensibilidade (92,5%), especificidade (97,1%) e acurácia (97%), o que confere robustez à sua utilização e à monitorização desse agravo.

Os critérios definidos por Silva *et al*.^2^ foram validados pelos estudos conduzidos por Kale *et al*.^3^ e França *et al*.^4^ No primeiro estudo^3^, uma coorte de nascidos vivos em duas capitais brasileiras, foram utilizados três critérios pragmáticos de Silva *et al*.^2^ para a definição do *near miss* neonatal: peso ao nascer < 1.500g, idade gestacional < 32 semanas e escore de Apgar < 7 no quinto minuto de vida. No segundo estudo^4^, também uma coorte de nascidos vivos, foram utilizados dados dos Sistemas de Informação em Saúde, selecionando-se as variáveis utilizadas no estudo de Kale *et al*.^3^, acrescidas de internação em unidade de terapia intensiva (UTI) neonatal e de más-formações congênitas. Ambos os estudos evidenciaram a acurácia da proposta de Silva *et al*.^2^

O indicador *near miss* neonatal possui inúmeras vantagens por ser uma ferramenta para identificar fatores de risco associados ao óbito neonatal e monitorar mudanças na morbimortalidade neonatal^5^. Dentre essas vantagens, cita-se a identificação de morbidades graves e de suas causas primárias, o que pode reduzir o óbito neonatal e permite que o indicador seja usado em diversas configurações para identificar problemas no sistema de saúde – configurando-se como instrumento de gestão – e, se for o caso, para instituir ações corretivas^2^, redundando em melhoria da qualidade da assistência neonatal^5^.

Em relação aos óbitos infantis no Brasil, observa-se que esse desfecho ocorre preponderantemente no período neonatal (70%), sobretudo nas primeiras semanas de vida (54%)^6^. Logo, há uma redução da mortalidade infantil no período pós-neonatal (de 23,1 para 9,5 por mil nascidos vivos)^6^.

Diante disso, vários autores têm discutido a mortalidade neonatal^6^^,^^7^; no entanto, são escassos os estudos que se detiveram na análise dos principais fatores associados ao *near miss* neonatal^5^^,^^8^. O avanço no conhecimento da rede de fatores de risco maternos envolvidos na mortalidade neonatal (idade, escolaridade^6^, situação conjugal, hábito de fumar e uso do álcool^9^, doenças prévias e atuais da gestação, adequação do pré-natal, entre outros)^10^, com base na estratégia de modelagem hierárquica para discriminar as relações entre determinantes do *near miss* neonatal, pode ser útil na sua avaliação; outrossim, possibilita apontar ações necessárias ao aperfeiçoamento da assistência, com consequente impacto nos desfechos neonatais.

Portanto, este estudo tem como objetivo avaliar a associação entre os fatores sociodemográficos, as características maternas, a organização dos serviços de saúde e o *near miss* neonatal em hospitais públicos e privados representativos das cinco regiões do Brasil.

## MÉTODOS

Esta pesquisa consiste em uma coorte prospectiva de nascidos vivos constituída de informações dos questionários aplicados às puérperas e de dados coletados dos prontuários de pacientes que participaram da pesquisa *Nascer no Brasil*. A coleta de dados ocorreu entre fevereiro de 2011 e outubro de 2012. Detalhes em relação à amostra são encontrados no estudo de Vasconcellos *et al*.^11^ e, sobre o método, em Leal *et al*.^12^

Para a construção da variável dependente deste estudo, o *near miss* neonatal, foi utilizada a classificação do estudo de Silva *et al*.^2^, que selecionou cinco variáveis associadas à mortalidade neonatal: peso ao nascer < 1.500g, índice de Apgar < 7 no quinto minuto de vida, uso de VM, idade gestacional < 32 semanas e presença de más-formações congênitas. Sendo assim, todos os RN que sobreviveram ao período neonatal e apresentaram pelo menos um dos preditores citados foram considerados casos de *near miss* neonatal^2^.

Foram amostrados 24.200 RN, sendo 23.837 deles nascidos vivos, 128 natimortos, 171 óbitos neonatais e 64 óbitos neonatais resgatados do Sistema de Informações sobre Mortalidade (SIM). Os casos de óbitos neonatais após a alta hospitalar foram obtidos por meio de um questionário aplicado após o 42° dia de internação da mulher ou no 28° dia de internação do RN. Informações mais detalhadas sobre o método podem ser obtidas no estudo de Silva *et al*^2^.

O modelo hierárquico do *near miss* neonatal foi baseado em fatores de risco para o óbito do RN^13^. Ressalta-se que as condições de saúde do RN e da atenção neonatal são inerentes à definição do *near miss* neonatal (idade gestacional, peso ao nascer, escore de Apgar, entre outros). Por isso, foram consideradas no nível proximal as variáveis relativas à organização do serviço de saúde. As variáveis independentes foram organizadas por nível de proximidade com o desfecho, inserindo-se primeiro as do nível distal e, em seguida, as dos níveis intermediário e proximal, estabelecidas a partir da literatura^1^^,^^2^^,^^6^^,^^8^ e organizadas em um modelo teórico conceitual (Figura).

**Figura:** Modelo teórico-conceitual dos fatores preditores do *near miss* neonatal no Brasil.

No nível distal, foram incluídos os aspectos sociodemográficos: região (Sudeste, Norte, Nordeste, Centro-Oeste ou Sul); escolaridade materna em anos completos (ensino fundamental incompleto, ensino fundamental completo, ensino médio completo ou ensino superior completo); classe econômica (A+B, C ou D+E); cor da pele (branca ou preta/parda/amarela/indígena); e chefe de família (não ou sim). Foi considerada “chefe de família” quando a mãe era a pessoa de referência para tomada de decisões na família^11^, e a classificação econômica foi baseada nos critérios da Associação Brasileira de Empresas de Pesquisa (Abep)^14^.

No nível intermediário foram incluídas as variáveis representativas das características maternas: idade materna (12 a 19 anos, 20 a 34 anos, ou maior ou igual a 35 anos); situação conjugal (sem companheiro ou com companheiro); primiparidade (não ou sim); tipo de parto (vaginal, com fórceps ou cesáreo); tabagismo materno, considerando o uso habitual de fumo após o quinto mês da gravidez (não ou sim); síndrome hipertensiva da gestação (não ou sim); sífilis (não ou sim); diabetes pré-gestacional (não ou sim); diabetes gestacional (não ou sim); suspeição de uso inadequado de álcool (não ou sim); e tipo de gestação (única ou gemelar). Para mensurar o tabagismo, a variável fumar após o quinto mês de gestação foi considerada de maior risco para o baixo peso no RN^15^. Quanto à suspeição de uso de álcool, utilizou-se o questionário T-ACE (acrônimo das palavras inglesas: *tolerance, annoyed*, *cut down* e *eye-opener*), composto por quatro questões principais, às quais é atribuída uma pontuação, sendo o valor máximo do questionário igual a cinco (a primeira questão vale até dois pontos e, da segunda à quarta pergunta, a valoração é de até um ponto). A pontuação total maior ou igual a dois indica um caso positivo, ou seja, a mãe é identificada como consumidora de álcool^16^. A variável síndrome hipertensiva da gestação se refere ao diagnóstico de hipertensão crônica, hipertensão gestacional, pré-eclâmpsia, eclâmpsia ou síndrome de Hellp^17^.

No nível proximal foram consideradas as variáveis relacionadas à organização do serviço de saúde: pré-natal adequado (não ou sim); tipo de serviço utilizado no pré-natal (público ou privado); e localização do hospital/maternidade (não capital ou capital). Considerou-se assistência pré-natal adequada aquela iniciada até a 12ª semana gestacional, com realização de no mínimo seis consultas (valor corrigido conforme idade gestacional no momento do parto), registro no cartão de pré-natal de pelo menos um resultado de cada exame de rotina e recebimento de orientação para maternidade de referência^10^.

A variável sexo do RN (masculino ou feminino) não foi incluída em nenhum nível de determinação hierárquica; porém, fez parte do modelo final por ser um importante preditor da mortalidade neonatal^7^.

Para a análise dos dados, inicialmente, foram estimadas as frequências absolutas e relativas das variáveis preditoras. A análise bivariada utilizou o teste qui-quadrado de Pearson, o risco relativo (RR) e intervalos de confiança (IC) a 95% para avaliar a associação das variáveis. A análise multivariada utilizou modelos de regressão de Poisson com variância robusta para identificar as variáveis associadas ao *near miss* neonatal. O RR foi utilizado para analisar a associação das variáveis sociodemográficas, maternas e da organização dos serviços de saúde com o *near miss* neonatal. As variáveis com valor-p < 0,20 na análise bivariada foram selecionadas para análise multivariada. Apenas as variáveis com valor-p < 0,05 no modelo multivariado foram mantidas no modelo final. As variáveis colineares com fator de inflação da variância <10 foram excluídas do modelo.

O estudo principal foi aprovado pelo Comitê de Ética em Pesquisa (CEP) da Escola Nacional de Saúde Pública da Fundação Oswaldo Cruz (Parecer n° 92/10; CAE: 0096.0.031.000-10). A presente pesquisa foi submetida ao CEP do Instituto Nacional de Saúde da Mulher, da Criança e do Adolescente Fernandes Figueira e aprovada pelo Parecer n° 3.376.235 (CAAE: 14248719.1.0000.5269), cumprindo os preceitos da Resolução n° 466/2012 do Conselho Nacional de Saúde^18^. Todas as mulheres participantes concederam suas entrevistas e informações por meio de um termo de consentimento livre e esclarecido.

## RESULTADOS

Nesta pesquisa, 832 foi o número ponderado de RN que cumpriram os critérios de *near miss* neonatal, e 23.005 não o fizeram, totalizando 23.837 RN. Na Tabela 1, observa-se que o risco de ocorrência de *near miss* neonatal, quando comparadas as categorias sociodemográficas, foi maior entre as mulheres que cursaram o ensino fundamental incompleto (4,2%), que se autodeclararam pretas/pardas/amarelas/indígenas (4,1%) e que pertenciam à classe econômica C (3,8%).

**Tabela 1:** Distribuição das condições sociodemográficas (nível distal) segundo *near miss* neonatal. Brasil, 2011–2012.

Variáveis		% Total	% NMN	RR	IC95%	p^a^
Sexo						
	Masculino	51,7	4,1	1,15	0,98–1,35	0,078
	Feminino	48,3	3,5	1	-	-
Distal						
Região						
	Sudeste	42,6	4,3	1,38	0,99–1,92	0,057
	Norte	9,5	3,1	0,99	0,63–1,56	0,980
	Nordeste	28,8	3,5	1,12	0,74–1,69	0,592
	Sul	12,5	3,1	1	–	–
	Centro-Oeste	6,6	4,1	1,31	0,86–2,01	0,204
Escolaridade						
	Ensino fundamental incompleto	26,5	4,2	1,18	0,82–1,69	0,357
	Ensino fundamental completo	25,6	3,6	1,03	0,69–1,52	0,875
	Ensino médio completo	38,9	3,7	1,04	0,68–1,59	0,837
	Ensino superior completo ou mais	8,9	3,5	1	–	–
Raça/cor						
	Branca	33,8	3,3	1	–	–
	Preta/parda/amarela/indígena	66,2	4,1	1,24	0,98–1,56	0,075
Chefe de família						
	Não	89,6	3,9	1	–	–
	Sim	10,4	3,0	0,76	0,57–1,02	0,071
Classe econômica^b^						
	Classe D+E	23,6	3,8	1,14	0,89–1,47	0,303
	Classe C	52,0	4,0	1,18	0,89–1,56	0,249
	Classe A+B	24,3	3,4	1	–	–

NMN: *near miss* neonatal; RR: risco relativo; IC95%: intervalo de confiança de 95%.

a Teste qui-quadrado de Pearson.

b Conforme classificação da Abep.

Quanto às características maternas (Tabela 2), o risco de *near miss* foi maior entre as mulheres que tiveram parto cesáreo (4,3%), que declararam fazer uso de fumo (5,1%), que tiveram síndrome hipertensiva da gestação (8,7%), diabetes pré-gestacional (12%) e gestacional (4,4%). Além disso, foi observado maior risco de *near miss* neonatal nas mulheres com gravidez gemelar (11,8%), quando comparadas às de gestação única (3,7%).

**Tabela 2:** Distribuição das características maternas (nível intermediário) segundo *near miss* neonatal. Brasil, 2011–2012.

Variáveis		% Total	% NMN	RR	IC95%	p*
Sexo						
	Masculino	51,7	4,1	1,15	0,98–1,35	0,078
	Feminino	48,3	3,5	1	-	-
Intermediário						
Idade (anos)						
	12 a 19	19,1	4,3	1,24	0,95–1,62	0,107
	20 a 34	70,5	3,4	1	–	–
	≥ 35	10,4	5,2	1,51	1,23–1,85	< 0,001
Situação conjugal						
	Sem companheiro	18,5	4,3	1,18	0,94–1,46	0,138
	Com companheiro	81,5	3,7	1	–	–
Primípara						
	Não	53,3	3,6	1	–	–
	Sim	46,7	4,0	1,12	0,94–1,35	0,207
Tipo de parto						
	Vaginal	46,6	3,2	1	–	–
	Fórceps	1,4	5,4	1,70	0,88–3,31	0,115
	Cesariana	52,0	4,3	1,36	1,10–1,67	0,004
Uso habitual de fumo						
	Não	92,8	3,7	1	–	–
	Sim	7,2	5,1	1,38	0,97–1,96	0,069
Síndromes hipertensivas gestacionais						
	Não	89,0	3,2	1	–	–
	Sim	11,0	8,7	2,71	2,21–3,33	< 0,001
Sífilis						
	Não	99,0	3,8	1	–	–
	Sim	1,0	4,9	1,31	0,65–2,64	0,448
Diabetes pré-gestacional						
	Não	99,0	3,7	1	–	–
	Sim	1,0	12,0	3,23	1,9–5,3	< 0,001
Diabetes gestacional						
	Não	91,8	3,8	1	–	–
	Sim	8,2	4,4	1,18	0,92–1,52	0,197
Suspeição de uso inadequado do álcool						
	Não há suspeita	3,9	4,3	1,16	0,66–2,03	0,609
	Suspeita de uso	10,0	4,5	1,23	0,94–1,61	0,126
	Não ingeriu álcool	86,1	3,7	1	–	–
Tipo de gestação						
	Única	98,8	3,7	1	–	–
	Gemelar	1,2	11,8	3,18	2,25–4,50	< 0,001

NMN: *near miss* neonatal; RR: risco relativo; IC95%: intervalo de confiança de 95%.

* Teste qui-quadrado de Pearson.

No bloco referente à organização dos serviços de saúde (Tabela 3), foi identificado maior risco de *near miss* neonatal nos filhos das mulheres que não realizaram o pré-natal adequado. Ademais, esse desfecho foi maior (5,5%) quando o parto ocorreu na capital.

**Tabela 3:** Distribuição da organização do serviço de saúde (nível proximal) segundo *near miss* neonatal. Brasil, 2011–2012.

Variáveis		% Total	% NMN	RR	IC95%	p*
Sexo						
	Masculino	51,7	4,1	1,15	0,98–1,35	0,078
	Feminino	48,3	3,5	1	-	-
Proximal						
Pré-natal adequado						
	Não	36,7	5,1	1,66	1,32–2,08	< 0,001
	Sim	63,3	3,1	1	–	–
Local das consultas pré-natais						
	Público	70,7	4,0	1,27	1,00–1,62	0,050
	Particular	29,3	3,1	1	–	–
Localização do hospital/maternidade						
	Não capital	63,4	2,8	1	–	–
	Capital	36,6	5,5	1,95	1,44–2,65	< 0,001

NMN: *near miss* neonatal; RR: risco relativo; IC95%: intervalo de confiança de 95%.

* Teste qui-quadrado de Pearson.

As Tabelas 1, 2 e 3 apresentam os resultados da análise bivariada para todas as variáveis independentes incluídas no modelo. No nível distal (Tabela 1), nenhuma variável foi associada ao desfecho. No nível intermediário (Tabela 2), foram associadas ao *near miss* neonatal as seguintes variáveis: idade maior ou igual a 35 anos (RR = 1,51; IC95% 1,23–1,85), parto cesáreo (RR = 1,36; IC95% 1,10–1,67), síndrome hipertensiva gestacional (RR = 2,71; IC95% 2,21–3,33), diabetes pré-gestacional (RR = 3,23; IC:95% 1,90–5,30) e gestação gemelar (RR = 3,18; IC95% 2,25–4,50). Não houve associação entre o diabetes *mellitus* gestacional e o *near miss* neonatal. No nível proximal (Tabela 3), foram associadas as seguintes variáveis: pré-natal não adequado (RR = 1,66; IC95% 1,32–2,08) e realização do parto na capital (RR = 1,95; IC95% 1,44–2,65).

A Tabela 4 apresenta o modelo de regressão multivariada. Verificou-se associação significativa entre o *near miss* neonatal e as seguintes variáveis: idade da mãe maior ou igual a 35 anos (RR = 1,32; IC95% 1,04–1,66), parto cesáreo (RR = 1,34; IC95% 1,07–1,67), uso habitual de fumo (RR = 1,48; IC95% 1,04–2,10), síndrome hipertensiva da gestação (RR = 2,49; IC95% 1,98–3,14), diabetes pré-gestacional (RR = 2,63; IC95% 1,36-5,05), gestação gemelar (RR = 2,98; IC95% 1,90–4,68), pré-natal não adequado (RR = 1,71; IC95% 1,36–2,16) e localização do hospital/maternidade na capital (RR = 1,89; IC95% 1,40–2,55).

**Tabela 4:** Regressão multivariada das condições sociodemográficas, das características maternas e da organização dos serviços de saúde segundo *near miss* neonatal. Brasil, 2011–2012.

Variáveis		RR ajustado	IC95%	p*
Sexo do recém-nascido				
	Masculino	1,18	1,00–1,40	0,054
	Feminino	1	-	-
Distal				
Raça/cor				
	Branca	1	–	–
	Preta/parda/amarela/indígena	1,21	0,95–1,55	0,126
Intermediário				
Idade (anos)				
	12 a 19	1,28	0,98–1,67	0,073
	20 a 34	1	–	–
	≥ 35	1,32	1,04–1,66	0,020
Tipo de parto				
	Vaginal	1	–	–
	Fórceps	1,75	0,81–3,77	0,151
	Cesariana	1,34	1,07–1,67	0,009
Uso habitual de fumo				
	Não	1	–	–
	Sim	1,48	1,04–2,10	0,031
Síndromes hipertensivas gestacionais				
	Não	1	–	–
	Sim	2,49	1,98–3,14	< 0,001
Diabetes pré-gestacional				
	Não	1		
	Sim	2,63	1,36–5,05	0,004
Tipo de gestação				
	Única	1	–	–
	Gemelar	2,98	1,90–4,68	< 0,001
Proximal				
Pré-natal adequado				
	Não	1,71	1,36–2,16	< 0,001
	Sim	1	–	–
Localização do hospital/maternidade				
	Não capital	1	–	–
	Capital	1,89	1,40–2,55	< 0,001

RR: risco relativo; IC95%: intervalo de confiança de 95%.

* Teste qui-quadrado de Pearson.

## DISCUSSÃO

Os resultados revelaram a proeminência das características maternas (nível intermediário) na determinação do *near miss* neonatal, com importante contribuição das condições assistenciais recebidas no pré-natal (nível proximal), consideradas fatores passíveis de intervenção^10^.

A faixa etária materna igual ou superior a 35 anos, considerada fator de risco para inúmeros desfechos negativos relacionados aos RN^19^^,^^20^, teve sua associação ao *near miss* neonatal confirmada nesta pesquisa, corroborando outros estudos da literatura^2^^,^^8^. O estudo de coorte prospectiva de nascimento em seis maternidades brasileiras conduzido por Kale *et al*.^8^ observou que os RN de mães com idade materna avançada apresentaram quase duas vezes mais risco de *near miss* neonatal. As mulheres com idade superior a 35 anos têm maior frequência de resultados perinatais adversos quando comparadas às mulheres com idade entre 20 e 34 anos, com destaque para prematuridade, baixo peso ao nascer e baixo índice de Apgar^20^. Além disso, os filhos dessas mulheres correm maior risco de morrer no período neonatal devido às complicações obstétricas secundárias a doenças pré-existentes^21^.

O parto cesáreo se manteve associado à ocorrência de *near miss* neonatal neste estudo, resultado já apontado na literatura da área^2^^,^^5^^,^^8^. Silva *et al*.^2^, também na pesquisa *Nascer no Brasil*, observaram que a chance de *near miss* neonatal foi duas vezes maior entre as mulheres que realizaram cesáreas, ou seja, essa variável aparece como fator de risco para tal desfecho, mas também como fator de proteção para a mortalidade neonatal, dado que as crianças nascidas de parto vaginal apresentaram maior taxa de mortalidade neonatal. Assim, a via de parto em si não seria causa da ocorrência de complicações materno-fetais, mas sim a indicação clínica de cesariana. Para elucidar esse ponto, seria necessário investigar se a indicação de cesárea era intraparto, devido a complicações materno-fetais, ou eletiva, sem qualquer base clínica^5^.

A aplicação do modelo hierarquizado nesta investigação apontou que, dentre os fatores maternos analisados, o uso habitual de fumo após o quinto mês de gestação foi associado ao aumento do risco de *near miss* neonatal. Os efeitos adversos do fumo materno durante a gravidez incidem sobre o peso do RN. Todavia, o fumo é um dos determinantes modificáveis mais importantes para minimizar o risco de baixo peso ao nascer e de outros resultados perinatais adversos^15^. O impacto negativo do tabagismo materno durante toda a gestação no comprimento e no perímetro cefálico do RN indica que tal comportamento tem uma relação linear inversa com essas dimensões: quanto maior o tempo de gestação com exposição ao fumo, menores serão as medidas antropométricas do RN^22^. Esses achados foram apontados em uma coorte de base populacional com 8.621 nascidos vivos europeus, na qual se observou que, desde o início do segundo trimestre até o término da gravidez, os fetos de mulheres que continuaram a fumar pesaram menos em relação aos das não fumantes. Mais especificamente, a diferença de peso prevista nos filhos de mulheres fumantes na 20ª semana (IC95%) foi de −2,6g (−5,1 a −0,1), e na 40ª semana gestacional foi de −207g (−231 a −182)^15^.

Em relação às doenças crônicas, foi observado que as mulheres com síndrome hipertensiva da gestação apresentaram o dobro de risco de *near miss* neonatal. De forma semelhante, Oliveira *et al*.^23^ e Nardello *et al*.^19^, em estudos transversais em maternidades de Recife e Sergipe, respectivamente, observaram que a hipertensão gestacional foi fortemente associada a desfechos neonatais adversos. Apesar de inúmeros fatores e teorias sugeridas para explicar as possíveis causas desse agravo, a etiologia da síndrome hipertensiva na gestação ainda é pouco conhecida^17^^,^^24^; no entanto, seus efeitos têm sido associados a prematuridade, Apgar baixo e asfixia neonatal^25^.

Na mesma linha de raciocínio, também foi observada associação entre diabetes *mellitus* pré-gestacional e *near miss* neonatal. O aumento da prevalência de diabetes *mellitus* pré-gestacional e gestacional nos últimos anos pode ser justificado pela epidemia de obesidade, pelo aumento da idade materna e pela detecção precoce da doença, considerando a maior cobertura do pré-natal e a diminuição do ponto de corte do diagnóstico da diabetes gestacional (glicemia em jejum reduzida de 92mg/dL para 85mg/dL)^26^. Assim como a síndrome hipertensiva da gestação, a presença de diabetes *mellitus* na gestação também está associada a elevado risco de morbimortalidade neonatal^27^. Alguns estudos^25^^,^^27^ se detiveram sobre a avaliação da associação entre diabetes pré-gestacional e alguns desfechos neonatais, sobretudo prematuridade, anomalias congênitas – como más-formações cardiovasculares –, asfixia perinatal, angústia respiratória e complicações metabólicas (hipoglicemia, hipocalcemia, policitemia e hiperbilirrubinemia). Ainda que esses estudos não tenham avaliado o efeito da doença sobre o *near miss* neonatal, as citadas repercussões negativas do diabetes *mellitus* pré-gestacional sobre a saúde do RN mostram uma possível elucidação de seus efeitos sobre esse desfecho.

Houve um risco aproximadamente três vezes maior de *near miss* neonatal para gestação gemelar neste estudo. Tal resultado revela que o parto gemelar – condição rara que apresenta diversas particularidades e dificuldades, não apenas no manejo clínico, mas também na abordagem científica – ainda é considerado um desafio para o serviço de saúde e para as investigações sobre maiores riscos maternos e perinatais^28^. Vale destacar que a gestação gemelar aumenta a taxa de mortalidade perinatal de duas a três vezes, principalmente devido ao nascimento prematuro, à restrição de crescimento intrauterino, ao baixo peso ao nascer e à anóxia intraparto^29^^,^^30^. Portanto, é de extrema importância que haja atendimento pré-natal adequado visando resultados maternos e perinatais melhores nessa condição.

A falta de acesso e a qualidade do pré-natal são determinantes notáveis para a ocorrência de *near miss* neonatal^5^^,^^8^. Neste estudo, a não adequação do pré-natal (nível proximal) foi associada a esse desfecho, aumentando seu risco. Embora a assistência pré-natal no Brasil tenha alcançado cobertura praticamente universal, persistem desigualdades no acesso ao cuidado adequado^10^^,^^11^. Ressalta-se que o acompanhamento pré-natal possibilita a detecção e o tratamento precoce de afecções maternas pré-existentes e/ou iniciadas no período gestacional, bem como de alterações no concepto, reduzindo o risco de complicações obstétricas e morte neonatal devido a prematuridade, más-formações ou infecções congênitas, as quais constituem as causas mais frequentes de morte neonatal no mundo^9^^,^^10^. Vale informar que o número adequado de consultas pré-natais (seis ou mais) não garante por si só a qualidade da assistência materno-infantil, sendo necessário assegurar o início precoce do pré-natal (até a 12ª semana de gestação), a assistência por profissionais qualificados, a existência de recursos físicos e materiais adequados, a realização dos exames preconizados e o tratamento oportuno, caso necessário^17^^,^^21^^,^^25^^,^^26^.

Em relação à localização do hospital/maternidade para a realização do parto, observou-se que o risco de ocorrência de *near miss* neonatal quase dobrou entre os RN que nasceram nas capitais. Esse resultado pode ser parcialmente explicado pelo fato de as não capitais terem menor oferta de serviços especializados para atendimento de alto risco^2^, com condições menos adequadas ao atendimento de gestantes nesse quadro. Assim, as capitais ficam sendo referência para gestantes residentes em não capitais que apresentam complicações na gravidez^10^. Ressalta-se que a gravidade da doença parece ser um fator de confusão na associação entre hospital de nascimento e *near miss* neonatal. A alta disponibilidade de UTI neonatais e a precoce intervenção médica em grandes centros urbanos são fatores apontados por Silva *et al*.^2^ como possíveis justificativas para a maior ocorrência do *near miss* neonatal nas capitais. O presente estudo não teve como objetivo analisar a gravidade dos casos de *near miss* neonatal, impossibilitando, assim, apontar se a situação dos RN se agravava antes ou após o tratamento.

A principal limitação do estudo foi o fato de não ter considerado os hospitais com menos de 500 partos e os nascidos em domicílio. Destaca-se que o início de uma segunda versão da pesquisa *Nascer no Brasil* está previsto para 2020, mas até o presente momento não foram encontrados artigos com dados de *near miss* neonatal em nível nacional.

Todavia, este estudo apresenta como vantagem ter sido realizado a partir de uma base de dados hospitalar representativa da população brasileira. Além disso, oferece uma análise hierarquizada da determinação do *near miss* neonatal, com um amplo conjunto de variáveis para a avaliação da saúde das gestantes brasileiras, permitindo analisar as inter-relações envolvidas na rede de causalidade desse desfecho. Portanto, a identificação das variáveis que exercem maior impacto na ocorrência do *near miss* neonatal possibilita a adoção de medidas de prevenção e de intervenção na assistência pré-natal das gestantes, repercutindo na saúde de seus RN.

## CONCLUSÕES

Embora características passíveis de intervenção pelo aconselhamento – como o uso habitual de fumo – tenham se associado à ocorrência do *near miss* neonatal, outros fatores determinantes desse desfecho se referiam à prestação dos serviços e dos cuidados. Portanto, é de extrema importância enfatizar a adequação do pré-natal para a identificação das gestantes que necessitam de atenção mais especializada, com acompanhamento oportuno durante a gravidez, o parto e o pós-parto para a prevenção de condições perinatais que ameaçam a vida.
